# Ponatinib efficiently kills imatinib-resistant chronic eosinophilic leukemia cells harboring gatekeeper mutant T674I FIP1L1-PDGFRα: roles of Mcl-1 and β-catenin

**DOI:** 10.1186/1476-4598-13-17

**Published:** 2014-01-28

**Authors:** Yanli Jin, Ke Ding, Honglin Li, Mengzhu Xue, Xiaoke Shi, Chengyan Wang, Jingxuan Pan

**Affiliations:** 1 Department of Pathophysiology, Zhongshan School of Medicine, Sun Yat-sen University, Guangzhou, China; 2 Key Laboratory of Tropical Disease Control (Sun Yat-sen University), Ministry of Education, Guangzhou, China; 3 Key Laboratory of Regenerative Biology and Institute of Chemical Biology, Guangzhou Institute of Biomedicine and Health, Chinese Academy of Sciences, Guangzhou Science Park, Guangzhou, China; 4 Shanghai Key Laboratory of Chemical Biology, School of Pharmacy, East China University of Science and Technology, Shanghai, China; 5 State Key Laboratory of Ophthalmology, Zhongshan Ophthalmic Center Sun Yat-sen University, 54 Xianlie Nan Road, Guangzhou 510060, People’s Republic of China; 6 Collaborative Innovation Center for Cancer Medicine, State Key Laboratory of Oncology in South China, Sun Yat-Sen University Cancer Center, Guangzhou 510060, China

**Keywords:** PDGFRα, T674I mutant, Tyrosine kinase inhibitor, Imatinib, Resistance, Ponatinib, Apoptosis

## Abstract

**Background:**

T674I FIP1L1-PDGFRα in a subset of chronic eosinophilic leukemia (CEL) is a gatekeeper mutation that is resistant to many tyrosine kinase inhibitors (TKIs) (e.g., imatinib, nilotinib and dasatinib), similar to T315I Bcr-Abl. Therefore, novel TKIs effective against T674I FIP1L1-PDGFRα are needed. Ponatinib (AP24534) is a novel orally bioavailable TKI against T315I Bcr-Abl, but it is not clear whether ponatinib is effective against T674I FIP1L1-PDGFRα. The purpose of this study was to examine the effect of ponatinib on T674I FIP1L1-PDGFRα.

**Methods:**

Molecular docking analysis *in silico* was performed. The effects of ponatinib on PDGFRα signaling pathways, apoptosis and cell cycling were examined in EOL-1, BaF3 cells expressing either wild type (WT) or T674I FIP1L1-PDGFRα. The in vivo antitumor activity of ponatinib was evaluated with xenografted BaF3-T674I FIP1L1-PDGFRα cells in nude mice models.

**Results:**

Molecular docking analysis revealed that ponatinib could bind to the DFG (Asp-Phe-Gly)-out state of T674I PDGFRα. Ponatinib potently inhibited the phosphorylation of WT and T674I FIP1L1-PDGFRα and their downstream signaling molecules (e.g., Stat3, Stat5). Ponatinib strikingly inhibited the growth of both WT and T674I FIP1L1-PDGFRα-carrying CEL cells (IC_50_: 0.004–2.5 nM). It induced apoptosis in CEL cells with caspase-3-dependent cleavage of Mcl-1, and inhibited tyrosine phosphorylation of β-catenin to decrease its stability and pro-survival functions. In vivo, ponatinib abrogated the growth of xenografted BaF3-T674I FIP1L1-PDGFRα cells in nude mice.

**Conclusions:**

Ponatinib is a pan-FIP1L1-PDGFRα inhibitor, and clinical trials are warranted to investigate its efficacy in imatinib-resistant CEL.

## Introduction

Platelet-derived growth factor receptor α (PDGFRα) is a class III receptor tyrosine kinase with an extracellular domain, a single transmembrane domain, and a cytoplasmic tyrosine kinase domain [1]. Upon ligand binding, the activated receptor drives multiple downstream pathways such as signal transducer and activator of transcription (Stat), Src kinases, mitogen-activated protein kinases, and phosphatidylinositol-3 kinase to coordinate cell proliferation, differentiation, survival, adhesion, and cell migration [2]. Gain-of-function mutations in PDGFRα have been found in chronic myeloid leukemia (CML), gastrointestinal stromal tumors (GISTs) and chronic eosinophilic leukemia (CEL) [3]. A typical example is the fusion gene of FIP1-like 1 (FIP1L1)-PDGFRα created by an 800-kb cryptic interstitial deletion in chromosome 4q12, which is pathogenic for a subset of CEL [1]. FIP1L1-PDGFRα encodes a ligand-independent and constitutively active tyrosine kinase that is sensitive to imatinib [4-6]. However, acquired resistance to imatinib can occur because of point mutations in the ATP binding site (e.g., T674I and D842V) [7-9]. T674I FIP1L1-PDGFRα is a “gatekeeper” mutation: substitution of the gatekeeper threonine (T674) with a bulky amino acid (I) blocks access by imatinib and second-generation tyrosine kinase inhibitors (TKIs), such as nilotinib and dasatinib, to a hydrophobic pocket inside the ATP binding site [10]. This gatekeeper mutation is analogous to the T315I mutation in Bcr-Abl [1,5,11,12]. The prognosis is poor for CEL patients harboring T674I FIP1L1-PDGFRα although it is rare in incidence.

To search for novel TKIs to overcome imatinib resistance, midostaurin, EXEL-0862 and sorafenib have been investigated both in vitro and in vivo in cells harboring T674I FIP1L1-PDGFRα [2,9,13]. Thus far, the first clinical trial of sorafenib for T674I FIP1L1-PDGFRα-positive CEL showed a transient hematological response, but patients died of rapid emergence of additional sorafenib-resistant point mutations in FIP1L1-PDGFRα [7]. Independently, Metzgeroth et al. reported limited clinical activity of sorafenib and nilotinib in T674I FIP1L1-PDGFRα-positive CEL patients [14]. Therefore, imatinib-resistant CEL remains a therapeutic challenge.

There has been exciting recent advance in third-generation TKIs (ponatinib, HG-7-85-1 and DCC-2036) efficacious against the gatekeeper mutants [15-18]. In vitro screening assay has demonstrated that ponatinib, the first TKI effective against T315I Bcr-Abl, is also a potent inhibitor of KIT, PDGFRα, Flt3, Src, VEGFR and FGFR [15,19,20].

We investigated the molecular docking of ponatinib to T674I PDGFRα *in silico*. In vitro and in vivo study then confirmed that ponatinib is a potent inhibitor of CEL cells bearing wild-type (WT) or T674I FIP1L1-PDGFRα.

## Results

### Computer-simulated binding of ponatinib to the native or mutated PDGFRα kinase in DFG(Asp-Phe-Gly)-out state

To gain insights into the structural basis for ponatinib to bind to the ATP-binding site of T674I PDGFRα, we performed computer simulations of molecular docking between ponatinib and T674I PDGFRα: ponatinib bound to native or mutated PDGFRα with the same orientation in the DFG-out state (Additional file 1: Figure S1A). These findings were similar to those observed in the complex between ponatinib and T315I Abl [15,21]. The T674I gatekeeper mutation does not perturb the overall protein structure of PDGFRα, except that the large aliphatic side chain causes a steric hindrance that prevents the binding of imatinib but not ponatinib (data not shown). The imidazo [1,2b] pyridazine scaffold of ponatinib docks in the adenine binding pocket of T674I PDGFRα and forms one hydrogen bond with the backbone N atom of C677 in the hinge region (distance 3.16 Å) (Additional file 1: Figure S1B). The ethynylene linker makes VDW interactions with the side chain of I674 residue, and the methylphenyl group occupies the hydrophobic pocket behind the gatekeeper residue. The extended amide linker contacts T674I PDGFRα by 2 hydrogen bonds, one with the backbone N atom of D836 (distance 3.76 Å) in the DFG motif and the other with the side chain carboxyl of E644 (distance 3.09 Å) in the C helix. Consequently, the trifluoromethylphenyl group binds to the pocket in the DFG-out conformation, with the terminal methylpiperazion group oriented to a solvent-exposed region.

Ponatinib and WT PDGFRα have an interaction profile (Additional file 1: Figure S1C) similar to but slightly different from that in Additional file 1: Figure S1B. Specifically, the whole WT PDGFRα molecule turns more closely to the DFG motif, with the corresponding H-bond distance shortened to 2.92 Å. The *in silico* structural comparisons revealed the importance of the DFG-out state and the ethynylene linker in ponatinib in avoiding a steric clash imposed by the mutated gatekeeper residue I674.

### Ponatinib inhibits PDGFRα phosphorylation

**Figure 1:**
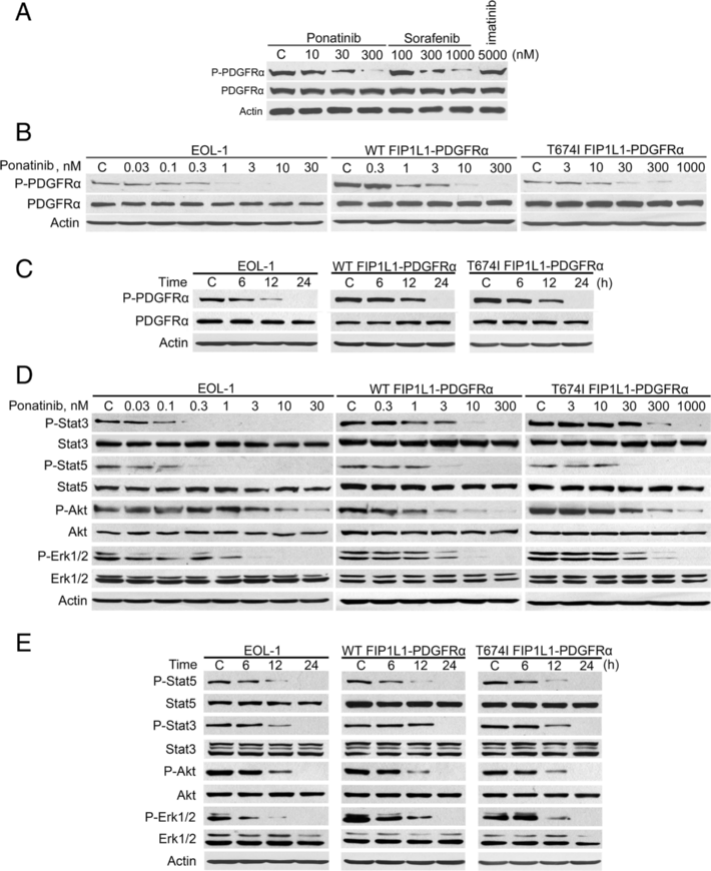
**Ponatinib inhibits phosphorylation of PDGFRα and its downstream signaling molecules. (A)** BaF3-T674I FIP1L1-PDGFRα cells exhibited differential sensitivity to ponatinib and sorafenib. BaF3-T674I FIP1L1-PDGFRα cells were treated with the TKIs at the indicated concentrations for 24 h, and the levels of phosphorylated and total PDGFRα were detected with the relevant antibodies. **(B)** Ponatinib inhibited phosphorylation of PDGFRα in a concentration-dependent manner. EOL-1 and BaF3-WT or -T674I FIP1L1-PDGFRα cells were exposed to escalating concentrations of ponatinib for 24 h. **(C)** Ponatinib inhibited phosphorylation of PDGFRα in a time-dependent manner. The concentrations of ponatinib were 1 nM for EOL-1, 300 nM for BaF3-WT and -T674I FIP1L1-PDGFRα cells, respectively. **(D)** Ponatinib concentration-dependently inhibited phosphorylation of Stat3, Stat5, Akt and Erk1/2. The cells were exposed to increasing concentrations of ponatinib for 24 h. **(E)** Ponatinib time-dependently inhibited phosphorylation of Stat3, Stat5, Akt and Erk1/2. 300 nM ponatinib was applied.

To examine whether ponatinib is active against T674I FIP1L1-PDGFRα, we exposed BaF3-T674I FIP1L1-PDGFRα cells to ponatinib, sorafenib and imatinib (the latter two serving as positive and negative controls, respectively); levels of phosphorylated and total PDGFRα were measured by immunoblotting. The phosphorylation of T674I FIP1L1-PDGFRα was altered by sorafenib but not imatinib (Figure 1A), which is consistent with a previous report [13]. In contrast to imatinib, 300 nM ponatinib inhibited phosphorylation of T674I FIP1L1-PDGFRα to a similar degree as 1000 nM sorafenib (Figure 1A). It also inhibited the phosphorylation of FIP1L1-PDGFRα in EOL-1, BaF3-WT FIP1L1-PDGFRα and BaF3-T674I FIP1L1-PDGFRα cells in concentration- and time-dependent manners (Figure 1B and C).

### Ponatinib inhibits downstream signaling of PDGFRα

We next examined signal transduction downstream of PDGFRα after ponatinib treatment. The phosphorylation of Stat3, Stat5, Akt and Erk1/2 were measured by immunoblotting with phospho-specific antibodies. Treatment for 24 h with ponatinib reduced the phosphorylation of Stat3, Stat5, Akt and Erk1/2 in cells expressing WT or T674I PDGFRα at 0.3-30 nM (Figure 1D) and over time (Figure 1E).

### Ponatinib inhibits growth of imatinib-resistant and -sensitive CEL cells bearing PDGFRα

**Figure 2:**
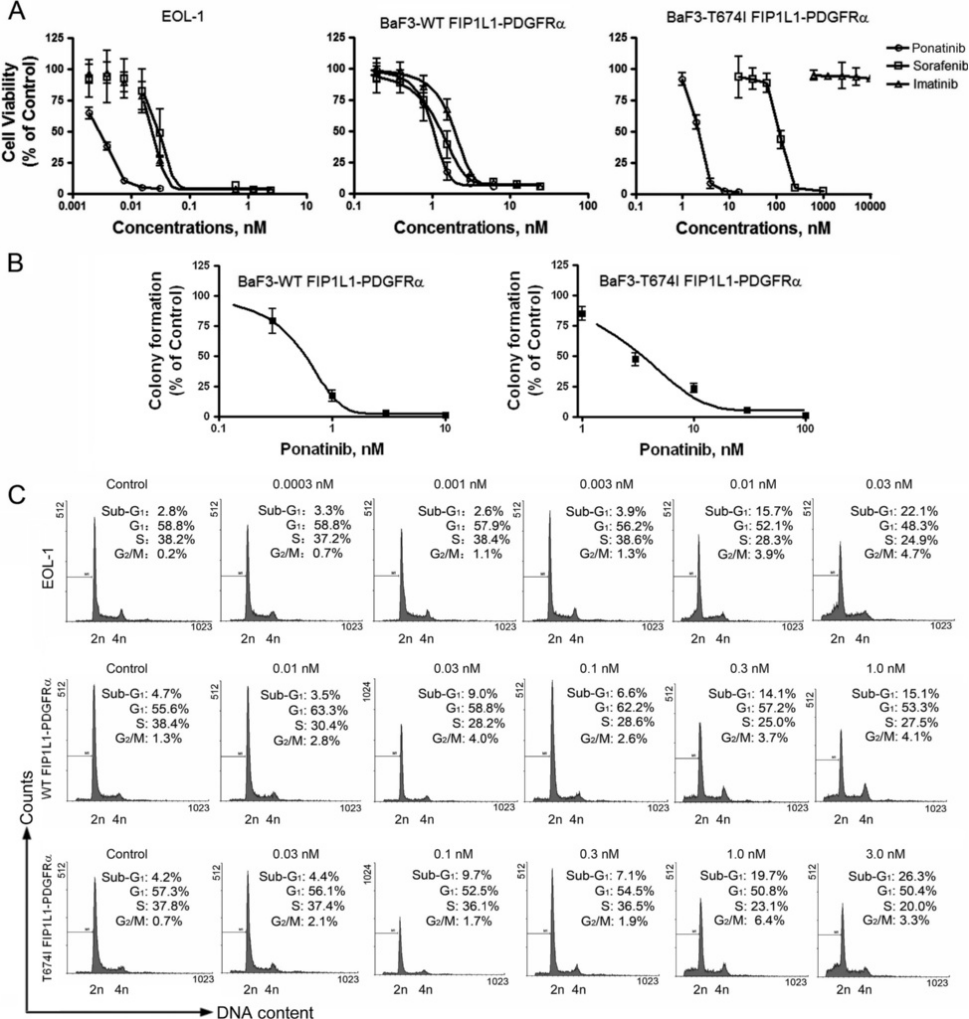
**Ponatinib inhibits the growth of neoplastic cells expressing PDGFRα. (A)** Ponatinib inhibited the cell viability of FIP1LI-PDGFRα-expressing cells. EOL-1 and BaF3-WT or -T674I FIP1L1-PDGFRα cells were exposed to increasing concentrations of ponatinib, sorafenib or imatinib for 72 h, and cell viability was evaluated by MTS assay. Graphs show data from 3 independent experiments; error bars represent 95% confidence intervals. **(B)** Clonogenicity of BaF3-WT or -T674I FIP1L1-PDGFRα cells was inhibited by ponatinib in a concentration-dependent manner. Error bars represent 95% confidence intervals. **(C)** Effect of ponatinib on cell cycle distribution in CEL cells. CEL cells were exposed to ponatinib for 24 h. Cells were fixed and analyzed by FACScalibur after staining with propidium iodide. Histograms are from representative experiments.

We examined the effect of TKIs on cell viability (MTS assay). The three lines of FIP1L1-PDGFRα-expressing cells were incubated with or without increasing concentrations of ponatinib, sorafenib, or imatinib for 72 h; log concentration-response curves are shown in Figure 2A. EOL-1 and BaF3-WT FIP1L1-PDGFRα cells were sensitive to imatinib, with IC_50_ values of 0.3 and 2.8 nM, respectively. BaF3-T674I FIP1L1-PDGFRα cells were resistant to imatinib, similar to previous reports [1,2]. Ponatinib inhibited the growth of all three FIP1L1-PDGFRα-expressing cell lines, with IC_50_ values of 0.004-2.5 nM. Notably, BaF3-T674I FIP1L1-PDGFRα cells were about 100-fold more sensitive to ponatinib than to sorafenib (IC_50_ = 2.5 versus 250 nM, respectively).

In another independent set of experiments, we evaluated the impact of ponatinib on clonogenicity of the two lines of BaF3 cells. Cells were exposed to increasing concentrations of ponatinib for 24 h, then equal numbers of treated cells were seeded in methylcellulose medium. Ponatinib concentration-dependently inhibited the number of clonogenic BaF3-WT or -T674I FIP1L1-PDGFRα cells (Figure 2B), with IC_50_ value 0.6 nM for BaF3-WT FIP1L1-PDGFRα and 2.8 nM for BaF3-T674I FIP1L1-PDGFRα cells.

Cell cycle distribution was analyzed by flow cytometry analysis of cellular DNA content after exposing the cells to increasing concentrations of ponatinib for 24 h. Ponatinib did not significantly change cell-cycle phase distribution except for an increase in sub-G_1_ particles, indicative of apoptosis (Figure 2C).

### Ponatinib induces apoptosis in both imatinib-sensitive and -resistant CEL cells by triggering the mitochondrial apoptosis pathway

**Figure 3:**
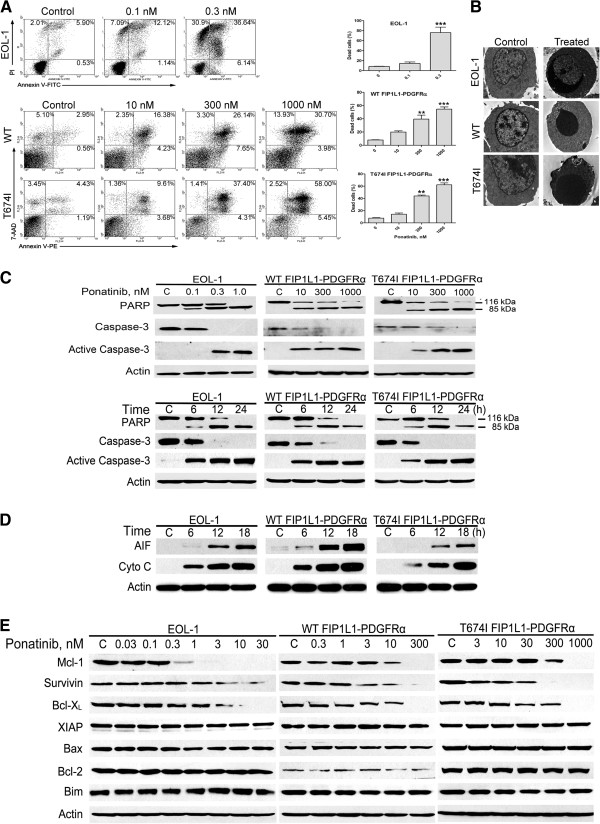
**Ponatinib induces apoptosis in FIP1LI-PDGFRα-expressing cells. (A)** EOL-1 and BaF3-WT or -T674I FIP1L1-PDGFRα cells were exposed to increasing concentrations of ponatinib for 24 h, apoptotic cells were assayed with flow cytometry by PI/Annexin V-FITC (EOL-1) or 7-AAD/Annexin V-PE (BaF3-WT or -T674I FIP1L1-PDGFRα cells) staining. Left, representative histograms; Right, statistical data of 3 independent experiments, the vertical axis stands for the sum of all dead cells. Error bars represent 95% confidence intervals. **, P < 0.01; ***, P < 0.0001, one-way ANOVA, *post hoc* comparisons, Tukey’s test. **(B)** The indicated cells were treated with or without ponatinib (1 nM for EOL-1, 300 nM for BaF3-WT and -T674I FIP1L1-PDGFRα cells, respectively) for 24 h, washed with PBS and fixed with 2% glutaraldehyde plus 2% paraformaldehyde in 0.1 M cacodylate buffer (pH 7.3). Representative photographs (9700×) were acquired under transmission electron microscopy. **(C)** The concentration- (for 24 h) and time-dependent (1 nM for EOL-1, 300 nM for BaF3-WT and -T674I FIP1L1-PDGFRα cells) cleavage of PARP and caspase-3 triggered by ponatinib was analyzed by immunoblotting. **(D)** Ponatinib elicited release of AIF and cytochrome *c* into the cytosol. Cells were treated with 1 nM ponatinib for the indicated durations and the cytosolic fraction was extracted with digitonin buffer. Levels of AIF and Cytochrome *c* (Cyto c) were detected by immunoblotting. **(E)** Immunoblotting of apoptosis-related proteins in CEL cells after treatment for 24 h.

We next assessed whether ponatinib induced apoptosis. The FIP1L1-PDGFRα-expressing cells were exposed to increasing concentrations of ponatinib for 24 h, and apoptosis was measured by Annexin V binding. Ponatinib led to remarkable apoptotic cell death in all 3 cell lines (Figure 3A). Further, condensation of chromatin in the periphery of the nuclei was observed by transmission electron microscopy, and this was consistent with the induction of apoptosis by ponatinib (Figure 3B). Moreover, ponatinib induced concentration- and time-dependent specific cleavage of PARP and caspase-3 activation in all three FIP1L1-PDGFRα-expressing cell lines (Figure 3C). Therefore, ponatinib could efficiently induce apoptosis in CEL cells harboring WT or T674I FIP1L1-PDGFRα.

To identify the apoptotic pathway triggered by ponatinib, the cells were treated with ponatinib, and AIF and cytochrome c in the cytosolic fraction were measured by immunoblotting. Ponatinib induced a time-dependent release of AIF and cytochrome c from the mitochondria into the cytosol (Figure 3D). In assessing the effect of ponatinib on the expression of apoptosis-related proteins, immunoblotting analysis revealed a prominent decrease in protein levels of Mcl-1, survivin and Bcl-X_L_, which are anti-apoptotic, with no effect on the levels of other apoptosis-related proteins such as XIAP, Bax, Bcl-2, and Bim (Figure 3E).

### Ponatinib elicits caspase-3-dependent cleavage of Mcl-1

**Figure 4:**
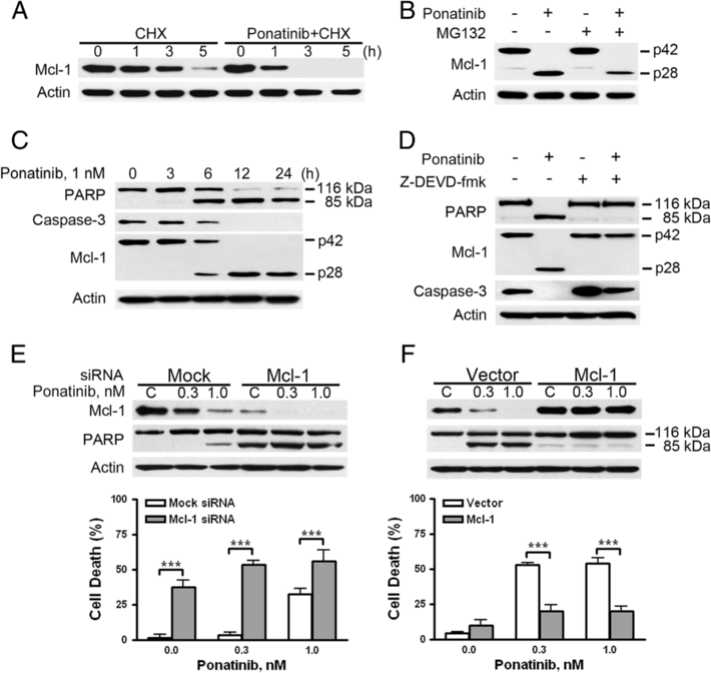
**Ponatinib mediates caspase-3-dependent cleavage of Mcl-1. (A)** Ponatinib precipitated in Mcl-1 turnover. After pretreatment with or without 1 nM ponatinib, EOL-1 cells were exposed to 5 μg/ml of cycloheximide (CHX), followed by Mcl-1 detection with immunoblotting. **(B)** MG-132 did not abrogate ponatinib-induced cleavage of Mcl-1. EOL-1 cells were treated with 1 nM ponatinib in the presence or absence of 0.5 μM MG-132 for 24 h. Mcl-1 level was then monitored with immunoblotting. **(C)** Mcl-1 cleavage occurred with onset of apoptosis after treatment with ponatinib. EOL-1 cells were treated with 1 nM ponatinib for different times, and the indicated proteins were measured with immunoblotting. **(D)** Mcl-1 cleaved in a caspase-3-dependent manner. EOL-1 cells were treated with 1 nM ponatinib for 24 h with or without 10 μM z-DEVD-fmk, then underwent immunoblotting. **(E)** Silencing Mcl-1 potentiated ponatinib-induced apoptosis in EOL-1 cells. Twenty-four hours after transfection with Mcl-1 siRNA or control (mock) siRNA, EOL-1 cells were treated with various concentrations of ponatinib, and levels of Mcl-1, PARP, and actin were evaluated by immunoblotting (top); parallel samples were examined for apoptosis by trypan blue staining (bottom, *** *P* < 0.0001, *t* test, error bars represent 95% confidence intervals; representative data from 3 independent experiments are shown). **(F)** Enforced overexpression of Mcl-1 abrogated the ponatinib-induced apoptosis. Twenty-four hours after transfection with pCMV5-flag empty vector or the plasmid expressing Mcl-1, EOL-1 cells were incubated with or without concentrations of ponatinib for another 24 h. Cell viability was evaluated by trypan blue dye exclusion (lower, *** *P* < 0.0001, *t* test, error bars represent 95% confidence intervals); Mcl-1 and PARP levels were detected by immunoblotting.

Because of the critical pro-survival role of Mcl-1 in leukemia [22,23], we explored its role in ponatinib-induced apoptosis of CEL cells. Mcl-1 mRNA levels did not significantly differ between ponatinib-treated CEL cells and controls (data not shown). Time chase experiments with inhibition of *de novo* protein synthesis by cycloheximide (CHX) revealed increased degradation of Mcl-1 level in ponatinib-treated CEL cells as compared with controls (Figure 4A). However, pretreatment with MG-132 (a specific proteasome inhibitor) did not prevent the ponatinib-induced degradation of Mcl-1 level (Figure 4B), which suggests that ponatinib decreased Mcl-1 level without involving proteasomes. Time-course study revealed that loss of Mcl-1 (p42) was accompanied by the appearance of a cleaved form Mcl-1 (p28) as apoptosis (specific cleavage of PARP) progressed (Figure 4C). These data are in agreement with a report of Mcl-1 being cleaved by caspase-3 at Asp^127^ to produce a 28-kDa fragment (Mcl-1^128-350^) [24]. To further confirm that ponatinib-induced Mcl-1 cleavage was caused by caspase-3 activation, CEL cells were treated with ponatinib in the absence or presence of the caspase-3 inhibitor z-DEVD-fmk. Immunoblotting revealed complete abrogation of decreased Mcl-1 level (p42) and appearance of the Mcl-1^128-350^ (p28) fragment (Figure 4D). Therefore, ponatinib-induced activation of caspase-3 may cleave and decrease the amount of Mcl-1.

Silencing Mcl-1 with specific siRNA duplex significantly enhanced the cytotoxicity of ponatinib, as reflected by PARP cleavage and cell death (Figure 4E). In contrast, enforced overexpression of Mcl-1 by transfection attenuated ponatinib-induced apoptosis in EOL-1 cells (Figure 4F). Taken together, cleavage of Mcl-1 by caspase-3 may form a positive feedback mechanism in the induction of apoptosis of CEL cells by ponatinib.

### Ponatinib inhibits tyrosine phosphorylation of PDGFRα

**Figure 5:**
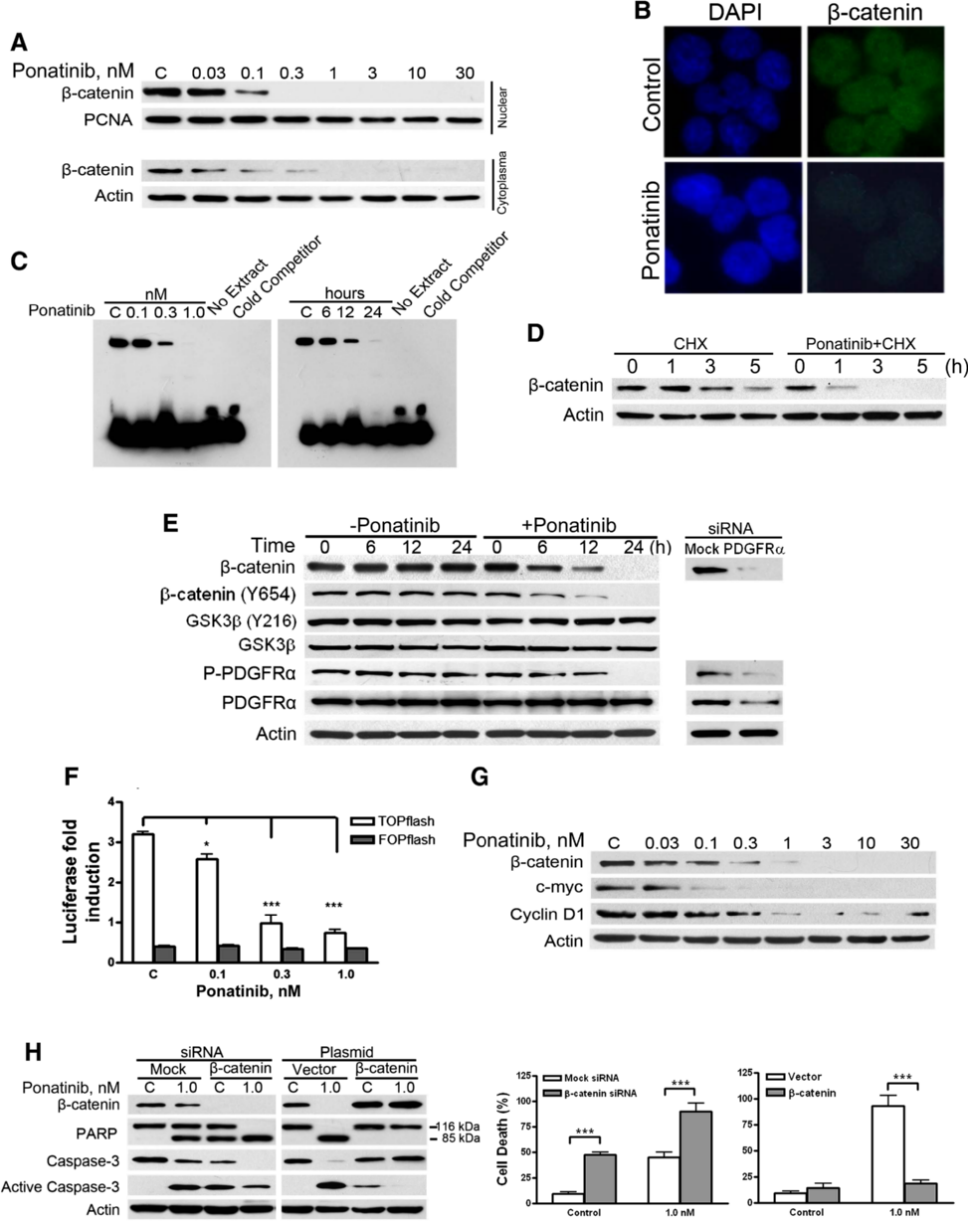
**Inhibition of tyrosine kinase activity of PDGFRα by ponatinib attenuates signaling of β-catenin by lowering its stability. (A)** Ponatinib concentration-dependently lowered β-catenin. EOL-1 cells were incubated with ponatinib for 24 h, and cytoplasmic and nuclear extracts were determined by immunoblotting. **(B)** Analysis of β-catenin localization. EOL-1 cells were pretreated with 1 nM ponatinib for 24 h, immunofluorescence analysis was performed with anti-β-catenin. Nuclei were stained with 4,6-diamidino-2-phenylindole (DAPI). **(C)** EOL-1 cells were pretreated with the indicated concentrations of ponatinib for 24 h or 1 nM ponatinib for various durations; and the nuclear extracts were then assayed for TCF/LEF activation by EMSA. **(D)** Ponatinib increased β-catenin turnover rate. After pretreatment with or without 1 nM ponatinib for 16 h, EOL-1 cells were exposed to 5 μg/ml of CHX, followed by immunoblotting for β-catenin. **(E)** Inhibition of PDGFRα decreased β-catenin. EOL-1 cells were treated with 1 nM ponatinib for various times, then total and tyrosine-phosphorylated β-catenin were evaluated (Left) by immunoblotting. EOL-1 cells were transfected with mock siRNA or PDGFRα siRNA, and β-catenin was monitored by immunoblotting. **(F)** Ponatinib abrogated TCF/LEF-dependent luciferase activity. EOL-1 cells were transfected with TOPflash and FOPflash plasmids and pEFRenilla-luc. After 24 h, the cells were treated with ponatinib for another 24 h, then underwent luciferase activity assay. **(G)** Ponatinib decreased the expression of target genes of β-catenin. Immunoblotting analysis in EOL-1 cells that were exposed to ponatinib for 24 h. **(H)** Ectopically changing the levels of β-catenin affected the ponatinib-mediated apoptosis. Twenty-four hours after transfection with control or Mcl-1 siRNA, or empty vector, pcDNA3-β-catenin, EOL-1 cells were treated with ponatinib, and the relevant protein levels were evaluated by immunoblotting (left); parallel samples were examined by the trypan blue dye exclusion assay (right, *** P<0.0001, *t* test; error bars represent 95% confidence intervals).

β-catenin, a critical effector in the canonical Wnt/β-catenin signaling cascade widely involved in cell proliferation, differentiation, escape of apoptosis and transformation [25], is a substrate of tyrosine kinases such as PDGFRα, Bcr-Abl, Flt3, and KIT [26-28]. Tyrosine phosphorylation of β-catenin leads to increased protein stability, keeping β-catenin active [26-29]. We therefore examined the potential change in tyrosine phosphorylation of β-catenin as a result of inhibition of PDGFRα by ponatinib. Because nuclear translocation of β-catenin is required for its functions (i.e., to activate TCF/LEF), we first examined whether ponatinib affected the subcellular distribution of β-catenin. With standard subcellular fractionation protocols, the levels of β-catenin in cytosolic and nuclear fractions were dose-dependently lowered by ponatinib (Figure 5A). Immunofluorescence analysis further confirmed that β-catenin was decreased by ponatinib in both cytosolic and nuclear compartments (Figure 5B). Electrophoretic mobility shift assay (EMSA) also revealed a concentration- and time-dependent decrease in nuclear β-catenin with ponatinib treatment (Figure 5C). Time chase experiments in the presence of CHX revealed that ponatinib led to an increased degradation rate of β-catenin (Figure 5D). Time-course study showed that the decrease in levels of β-catenin occurred concurrently with tyrosine dephosphorylation in β-catenin after inactivation of PDGFRα by ponatinib (Figure 5E). EOL-1 cells transfected with siRNA against PDGFRα displayed decreased levels in PDGFRα and β-catenin (Figure 5E, right), further supporting the specific effect of PDGFRα on β-catenin stability. These data support that tyrosine phosphorylation in β-catenin by PDGFRα directly promotes β-catenin stability.

### Ponatinib inhibits TCF/LEF-dependent reporter gene transcription

Nuclear β-catenin in complex with TCF/LEF transcription factors can activate target genes whose promoter contains the regulatory elements [30]. We therefore examined whether ponatinib treatment influenced TCF/LEF-dependent transcription. EOL-1 cells were cotransfected with TOPflash (or FOPflash) and pEFR*Renilla*-Luc for 24 h, cultured with or without ponatinib for an additional 24 h, then luciferase assay was performed. Ponatinib concentration-dependently inhibited the luciferase activity of TOP promoter constructs with optimized TCF-binding sites (Figure 5F). As a negative control, the luciferase activity of FOP promoter constructs with mutated TCF-binding sites was not changed.

### Ponatinib decreases the level of TCF/LEF-dependent genes involved in proliferation

We next examined whether ponatinib inhibited the expression of target genes (e.g. c-Myc and cyclin D1) of β-catenin-TCF/LEF. Immunoblotting revealed EOL-1 cells incubated with or without ponatinib for 24 h showing a concentration-dependent decrease in c-Myc and cyclin D1 levels (Figure 5G).

### β-catenin plays a significant role in ponatinib-induced apoptosis

EOL-1 cells transfected with specific siRNA duplex against β-catenin underwent remarkable apoptosis as compared with cells transfected with control siRNA, as reflected by PARP cleavage, caspase-3 activation and trypan-blue staining (Figure 5H, left). In addition, silencing β-catenin potentiated the sensitivity of EOL-1 cells to ponatinib (Figure 5H, left). Conversely, EOL-1 cells transfected with constructs encoding full-length β-catenin led to decreased sensitivity of EOL-1 cells to ponatinib (Figure 5H, right).

### Ponatinib inhibits growth of xenografted T674I FIP1L1-PDGFRα cells in nude mice

**Figure 6:**
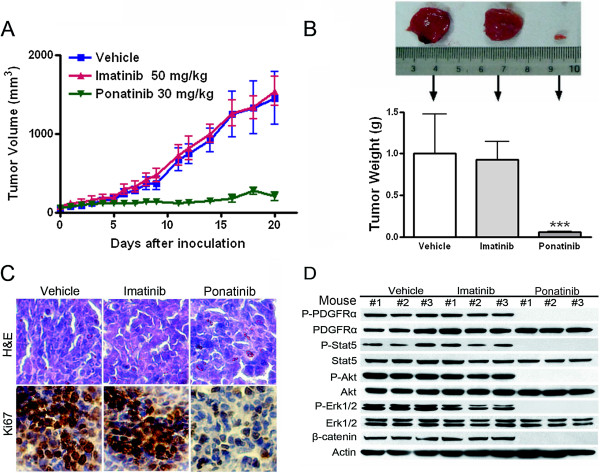
**Ponatinib potently abrogates the growth of imatinib-resistant neoplastic cells expressing T674I FIP1L1-PDGFRα in nude mouse xenografts. (A)** BALB/c *nu/nu* nude mice were subcutaneously inoculated with BaF3-T674I FIP1L1-PDGFRα cells, then randomized into 3 groups (10 animals each) for daily oral administration of vehicle [30% Cremophor EL/ethanol (4:1), 70% PBS], imatinib or ponatinib during days 5–21 after inoculation of cells. The tumor growth curves are plotted. Error bars represent 95% confidence intervals. **(B)** Dissected tumor xenografts were measured on day 21. ***, *P* < 0.0001, one-way ANOVA, *post hoc* comparisons, Tukey’s test. Columns, mean; error bars, 95% confidence intervals. Representative tumors removed from mice of each group are shown (*upper*). **(C)** Immnunohistochemical analysis with anti-Ki67 and H & E staining of xenograft tissues from mice sacrificed 21 days after tumor inoculation. **(D)** The signaling of PDGFRα in tumor tissue was inhibited by ponatinib. Whole cell lysates prepared from xenografts of each group were detected by immunoblotting with the indicated antibodies.

The in vivo antineoplastic activity of ponatinib as a single agent against imatinib-resistant T674I FIP1L1-PDGFRα-expressing cells was evaluated in the nude mouse xenograft model. Thirty *nu/nu* BALB/c mice were subcutaneously injected with BaF3-T674I PDGFRα cells. Four days later, when tumor sizes were ~50 mm^3^, the mice were randomized to receive treatment with vehicle, imatinib (50 mg/kg/d) or ponatinib (30 mg/kg/d) for 15 days (n = 10). The tumor growth curve (the estimated tumor size calculated from the tumor dimension versus time) with imatinib was almost the same as with vehicle (Figure 6A), which indicates in vivo resistance of BaF3-T674I PDGFRα cells to imatinib. In contrast, ponatinib treatment abrogated the growth of tumors (Figure 6A). Tumor weight did not differ between imatinib- and vehicle-treated tumors but was lower in ponatinib-treated than control tumors (Figure 6B). Cell proliferation, as reflected by Ki67 immunohistochemistry, was inhibited by ponatinib (Figure 6C) as compared with the two controls. Immunoblotting of cell lysates from tumors from ponatinib-treated mice showed pronounced decreases in phosphorylated PDGFRα, Stat5, Akt, and Erk1/2 but not their total counterparts, so ponatinib blocked PDGFRα signaling in xenografts (Figure 6D). In addition, the level of β-catenin was decreased by ponatinib (Figure 6D). The body weights of the mice remained stable, with no significant differences between drug-treated and control mice (data not shown). Motor activity and feeding behavior of the mice were all normal. No mice died until the scheduled sacrifice. Overall, ponatinib was well tolerated at the dosage used.

## Discussion

Acquired resistance to TKIs presents a therapeutic challenge. Gatekeeper mutants (e.g. T315I Bcr-Abl, T670I KIT and T674I PDGFRα) are particularly multi-drug resistant. In the present study, ponatinib potently inhibited the phosphorylation of the WT and gatekeeper mutant T674I FIP1L1-PDGFRα and their downstream signaling. Our molecular docking analysis revealed that ponatinib could target native or T674I FIP1L1-PDGFRα in the DFG-out (inactive) binding mode, similar to ponatinib docking in T315I Bcr-Abl. This characteristic of ponatinib may be related to its imidazo [1,2b] pyridazine core that occupies the pocket for adenine in the enzyme, whereas the methylphenyl group occupies the hydrophobic pocket behind the gatekeeper residue of the enzyme [15]. Encouraged by the *in silico* simulation results, we evaluated the efficacy of ponatinib against imatinib-resistant CEL cells both in vitro and in vivo.

Ponatinib potently inhibit the viability of EOL-1 cells expressing WT FIP1L1-PDGFRα, with an IC_50_ value of 0.004 nM. This efficacy agrees with recent results [20] showing an inhibitory effect in EOL-1 cells, with an IC_50_ of 0.5 nM. In the same study, ponatinib inhibited malignant cells expressing Bcr-Abl, Flt3, KIT, FGFR1, with IC_50_ values from 2 to 36 nM [20]. We showed that ponatinib had an inhibitory effect on imatinib-resistant leukemic BaF3-T674I FIP1L1-PDGFRα cells, with an IC_50_ of 2.5 nM, which is comparable to the potency in BaF3-T315I Bcr-Abl cells, with an IC_50_ of 11 nM [15]. Clonogenicity assay confirmed that ponatinib restrained the proliferation of BaF3-WT or -T674I FIP1L1-PDGFRα cells at low nanomolar concentrations. Further, our in vivo data revealed that ponatinib, at an oral dose of 30 mg/kg/day, potently abrogated the growth of xenografted imatinib-resistant BaF3-T674I FIP1L1-PDGFRα cells, with PDGFRα signaling highly suppressed (Figure 6). A pharmacokinetics study in mice indicated that orally administrated ponatinib as a single oral dose of 30 mg/kg, which was well tolerated, resulted in mean plasma concentrations of 782 and 561 nM at 2 and 6 h post-dosing, respectively [15]. Such plasma levels highly exceed the in vitro IC_50_ values for all 3 lines of FIP1L1-PDGFRα-expressing cells, so ponatinib may efficiently inhibit the growth of FIP1L1-PDGFRα-positive cells with clinically achievable doses.

Ponatinib induced remarkable apoptosis in both imatinib-sensitive and -resistant CEL cells, as evidenced by Annexin V binding, activation of caspase-3, and specific cleavage of PARP. The apoptosis was triggered by the mitochondrial-dependent pathway because of release of AIF and cytochrome c to the cytosol after treatment with ponatinib. The levels of survivin, Bcl-X_L_ and Mcl-1were decreased in ponatinib-mediated apoptotic CEL cells. The transcription of survivin and Bcl-X_L_ is regulated by Stat3, Stat5 and Erk1/2 [31,32]. The decreased expression of survivin and Bcl-X_L_ caused by ponatinib treatment is likely associated with the inhibition of Stat3, Stat5 and Erk1/2. However, future experiments can further define the precise mechanisms.

Mcl-1, a pro-survival and anti-apoptotic protein with relatively short-half life in the Bcl-2 family, is expressed in malignant hematological cells and protects cells against apoptosis in response to chemotherapeutic agents including TKIs [22,33]. The decrease in Mcl-1 by ponatinib in CEL cells may facilitate apoptosis, because silencing Mcl-1 with siRNA significantly potentiated the ponatinib-mediated apoptosis in EOL-1 cells, which is in line with the finding that decreased Mcl-1 level sensitizes leukemia cells to tyrosine kinase inhibitors [22]. Forced overexpression of Mcl-1 protected CEL cells against apoptosis in response to ponatinib. Regarding the mechanism underlying the decrease in Mcl-1 level by ponatinib, our results do not support the involvement of the ubiquitin-proteosome and transcription-dependent pathways. Rather, our data support a caspase-3-dependent mechanism, which is consistent with findings for other small-molecule tyrosine kinase inhibitors [9]. Of note, the resulting truncated form of Mcl-1^128–350^ (p28) cleaved by activated caspase-3 can potentiate apoptosis [24,34]. Mcl-1^128–350^ (p28) after ponatinib treatment likely produces a positive feedback to apoptosis. Although Bim has been reported as the primary death effector in TKIs-treated CML cells [35], no appreciable change was noted in ponatinib-treated CEL cells in the present study. However, because Mcl-1 has been demonstrated to neutralize Bim through complex formation to prevent apoptosis, our observed decline in Mcl-1 by ponatinib would increase the Bim/Mcl-1 ratio, which may release Bim to promote apoptosis [36].

The observed decrease in β-catenin induced by ponatinib exposure may be important because of the fundamental functions of β-catenin in cell proliferation, differentiation and apoptosis. [25]. Besides accumulating in a Wnt/GSK3β-dependent way, β-catenin may also accumulate after phosphorylation by tyrosine kinases (e.g., Bcr-Abl, RET, KIT, Flt3, PDGFRα) [26-28,37]. Inactivating PDGFRα by treating EOL-1 cells with ponatinib inhibited tyrosine phosphorylation (Y654) and concurrently decreased levels of β-catenin, as reflected by immunoblotting and immunofluorescent staining (Figure 5). Silencing PDGFRα also lowered β-catenin level, which further supports the specificity of the effect of PDGFRα on the levels of β-catenin. The turnover rate is enhanced in EOL-1 cells pretreated with ponatinib. Of note, inactivation of PDGFRα by ponatinib decreased β-catenin level in both cytosolic and nuclear pools. Ponatinib also decreased β-catenin level in xenografts in in vivo experiments.

The decrease in β-catenin level by ponatinib has functional consequences. First, TCF/LEF-dependent gene transcription was impaired in EOL-1 cells treated with ponatinib. Second, the expression of β-catenin-dependent genes such as c-Myc and cyclin D1 was decreased. Third, the binding of β-catenin and DNA was also decreased, as revealed by EMSA. Because β-catenin plays a crucial role in controlling self-renewal and differentiation in both normal and cancer stem cells [25], a decrease in β-catenin level may be an important aspect of the antineoplastic mechanism of ponatinib. An interesting hypothesis for future research is whether decreased β-catenin can facilitate the eradication of cancer stem cells.

While this manuscript was under review, Lierman et al. reported that ponatinib was active against imatinib-resistant FIP1L1-PDGFRα mutants [38]. Our results corroborated and extended their findings by providing a mechanism for the induction of apoptosis and evidence for in vivo efficacy.

## Conclusions

In conclusion, ponatinib is a potent inhibitor against both WT and T674I FIP1L1-PDGFRα. Caspase-3-dependent Mcl-1 cleavage may be a positive feedback mechanism to enhance apoptosis in ponatinib-treated cells. Inhibition of PDGFRα activity by ponatinib leads to decreased tyrosine phosphorylation of β-catenin, decreased protein stability and protein level of β-catenin, decreased transcription of TCF/LEF-regulated genes, and enhanced cytotoxicity. Therefore, regulation of β-catenin by PDGFRα plays a role in the antineoplastic mechanism of ponatinib. Given the FDA approval of oral ponatinib in patients with refractory CML and Ph + ALL resistant to the first- and second-generation of TKIs [39], our findings warrant a clinical trial of ponatinib in imatinib-resistant CEL and other malignant disorders harboring T674I PDGFRα.

## Materials and methods

### Reagents

Ponatinib (purity > 95%, HPLC) was synthesized in our lab. Imatinib and sorafenib were purchased from Alexis Biochemicals (Plymouth Meeting, PA). 4′, 6-diamidino-2-phenylindole (DAPI) was from Invitrogen. Cycloheximide (CHX) and propidium iodide (PI) were from Sigma-Aldrich. TOPflash/FOPflash system consisting of optimized TCF binding sites (TOP) or mutated sites (FOP) controlling the expression of a luciferase reporter gene was from Upstate Biotechnology (Lake Placid, NY). pCMV5-flag-human Mcl-1 and pcDNA3-β-catenin were kindly provided by Dr. Mien-Chie Hung (The University of Texas MD Anderson Cancer Center, Houston, TX) [40]. ON-TARGETplus SMARTpool small interfering RNA (siRNA) duplexes against human Mcl-1 or PDGFRα, and Non-Targeting Pool siRNA control were from Dharmacon RNA Tech. (Lafayette, CO) [8,41].

### Cell culture and cell growth measurement

The EOL-1 cell line harboring the FIP1L1-PDGFRα fusion oncogene was purchased from DMSZ (Braunschweig, Germany). BaF3 cells expressing WT or T674I FIP1L1-PDGFRα were cultured as described previously [8,9].

Cell viability was assessed by MTS assay (CellTiter 96 Aqueous One Solution reagent, Promega, Shanghai) [40,42].

Clonogenicity assay was performed as described [40]. In brief, 2×10^5^/ml cells were treated with drugs or diluent (DMSO, control) for 24 h, then washed with PBS and seeded in methylcellulose medium (Methocult M3231, Stem Cell Technologies, Vancouver, Canada) [40]. After incubation for ~7 days at 37°C and 5% CO_2_, colonies with >50 cells were counted [40].

### Preparation of whole cell lysates and cytosolic fraction

Most experiments of immunoblotting involved whole lysates prepared with RIPA buffer unless otherwise stated [42,43]. To measure the levels of AIF and cytochrome *c* in the cytosol, the cytosolic extract was prepared with digitonin extraction buffer [42,43].

### Preparation of cytoplasmic and nuclear fractions

Distribution of β-catenin was determined in the cytoplasmic and nuclear fractions as we previously described [44].

### Immunoblotting

Immunoblotting involved use of whole cell lysates prepared in RIPA buffer [8,9,40]. Antibodies and their sources were as follows: antibodies against apoptosis-inducing factor (AIF), Mcl-1 (S-19), Bax and Bcl-X_L_ (Santa Cruz Biotechnology, Santa Cruz, CA); antibodies against poly(adenosine diphosphate [ADP]-ribose) polymerase (PARP), X-linked inhibitor of apoptosis (XIAP), caspase-3, active caspase-3, cytochrome c (clone 6H2.B4), survivin, and C-terminal β-catenin (BD Biosciences Pharmingen, San Jose, CA); phospho-β-catenin (Y654) (Abcam, Cambridge, MA); antibodies against phospho-PDGFRα (Y1018), phospho-Erk1/2 (T202/Y204) and Erk1/2 (Cell Signaling Technology, Beverly, MA); antibodies against phospho-Stat3 (Y705), phospho-Stat5A/B (Y694/Y699), Stat3, Stat5, Bcl-2 and PDGFRα (Upstate Technology, Lake Placid, NY); anti-Bim (Stressgen, Ann Arbor, MI); anti-actin (Sigma-Aldrich, Shanghai). Anti-mouse immunoglobulin G and anti-rabbit immunoglobulin G horseradish peroxidase-conjugated antibodies were from Pierce Biotechnology (Rockford, IL, USA).

### Plasmids or small interfering RNA transfection

EOL-1 cells were transfected with plasmids or siRNA duplexes with use of Nucleofector (Amaxa, Gaithersburg, MD) by use of the Cell Line Nucleofector Kit T (Amaxa) and program O-17 [8]. At 24 h after transfection, EOL-1 cells were adjusted to 2×10^5^/ml and exposed to ponatinib treatment, then underwent cell death assay and immunoblotting.

### Luciferase assay

EOL-1 cells (2×10^5^) were transfected with TOPflash or FOPflash plasmid (0.5 μg) and pEF*Renilla*-luc (10 ng) by electroporation. At 24 h, cells were incubated with or without ponatinib for 24 h. Luciferase activity was then measured with the dual-luciferase assay kits (Promega, Shanghai) as described [45].

### Electrophoretic mobility shift assay (EMSA)

EMSA involved the LightShift Chemiluminescent EMSA kit (Pierce Biotechnology, Shanghai). The oligonucleotides for TCF/LEF were from Promega (Shanghai) with sequences as described [46]: forward, 5-TGCCGGGCTTTGATCTTTG-3; reverse, 5-AGCAAAGATCAAAGCCCGG-3. In brief, oligonucleotides for TCF/LEF were labeled with biotin by use of the biotin 3′-end DNA labeling kit (Pierce Biotechnology, Shanghai). In total, 5 μg of nuclear extracts was incubated for 20 mins with 1 μg/μl poly(dI-dC) and biotin end-labeled target nucleotides in 20-μl reaction mixtures. The resulting bound complex was separated from free oligonucleotides on 6% native polyacrylamide gel and transferred to a nylon membrane. After cross-linking, blocking, and reacting with substrates, the membranes were exposed to X-ray film to detect biotin-labeled DNA. The binding specificity was examined by competition with a 200-fold excess of the unlabeled oligonucleotide probe (cold competitor) [40,47].

### Transmission electron microscopy

The cells were treated with or without ponatinib, and then fixed with 2% glutaraldehyde plus 2% paraformaldehyde in 0.1 M cacodylate buffer (pH 7.3). After washing, and postfixing, the samples were dehydrated and embedded in Spurr’s low-viscosity medium [48]. Ultrathin sections of the samples stained with uranyl acetate and lead citrate were examined under a JEM 1010 transmission electron microscope [48].

### Immunofluorescence staining

EOL-1 cells were treated with or without ponatinib for 24 h, and then harvested by use of Cytospin onto glass slides. Immunofluorescence staining was as described [42]. DyLight 488 conjugated-goat-anti-mouse immunoglobulin was purchased from Pierce Biotechnology (Rockford, IL).

### Apoptosis assessment

Apoptosis was evaluated by using an Annexin V-fluorescein isothiocyanate (for EOL-1) or Annexin V-phycoerythrin (for BaF3 cells expressing FIP1L1-PDGFRα) apoptosis detection kit (BD Biosciences Pharmingen, San Jose, CA) and analyzed by using a FACSCalibur flow cytometer [8,9,40].

### Cell cycle analysis

Control or ponatinib-treated cells were fixed with 66% ethanol overnight. DNA content was analyzed by flow cytometry after cells were stained with 50 μg/ml PI and 2.5 μg/ml RNase in PBS solution for 30 mins [42,43].

### Tumor xenograft experiments

Male *nu/nu* BALB/c mice were bred at the animal facility of Sun Yat-sen University. An amount of 1×10^7^ BaF3-T674I PDGFRα cells supplemented with 50% matrigel was inoculated subcutaneously on the flanks of 4~6-week-old male nude mice. Tumors were measured every other day with calipers. Tumor volumes were calculated by the following formula: *a*^2^ × *b* × 0.4, where *a* is the smallest diameter and *b* is the diameter perpendicular to *a*. Ponatinib was initially dissolved in DMSO and then adjusted to the appropriate doses with vehicle [30% Cremophor EL/ethanol (4:1), 70% PBS], and imatinib was dissolved in sterile double-distilled water. Mice in each group were treated once daily by oral gavage with ponatinib, imatinib or the same amount of vehicle. The body weight, feeding behavior and motor activity of each animal were monitored as indicators of general health. Tumor xenografts were immediately removed, weighed, stored and fixed after animals were killed. All animal studies were conducted with the approval of the Sun Yat-sen University Institutional Animal Care and Use Committee.

### Immunohistochemical staining

Formalin-fixed BaF3-T674I FIP1L1-PDGFRα-cell xenografts were embedded in paraffin, sectioned (4-μm thick), then immunohistochemically stained by using the anti-Ki67 MaxVision kit (Maixin Biol, Fuzhou, China). Color was developed with 0.05% diaminobenzidine and 0.03% H_2_O_2_ in 50 mM Tris–HCl (pH 7.6), and slides were counterstained with hematoxylin [47].

### Homology modeling

The kinase domain sequence was identified from the Human Kinome database [49] by sequence alignment of the kinase domain within the full-length PDGFR*α* sequence (NCBI protein database, GI: 1736333) and the site to be mutated by use of CLUSTAL X [50].

Prime module in Maestro (Schrödinger Inc., v7.5) was chosen to build homology models for the native kinase domain sequence and the mutated sequence. We performed a BLAST search against the PDB database to choose a suitable template; the 1.6 Å X-ray structure of KIT kinase (PDB code: 1 T46) in complex with imatinib was chosen (identity 61%, E-value 4.1e-65) [51]. After SSP modification and alignment editing in Prime, native and mutated kinase models were built with default parameter sets, followed by loop refinement and energy minimization to eliminate and correct disallowed torsion angles and unfavorable atom-atom conflicts.

### Molecular docking

The compound ponatinib was prepared by the Ligprep module, then the Glide module. Then docking simulations were performed to test binding of ponatinib to the native kinase model and the mutated one with the same default parameter sets. The grid-enclosing box was centered on the centroid of the aligned ligand (imatinib) and defined to enclose residues located within 20 Å around the ATP binding site; a scaling factor of 1.0 was set to van der Waals (VDW) radii of the receptor atoms with the partial atomic charge < 0.25. The Extra-Precision (XP) mode of Glide was used to dock ponatinib into the ATP binding site with default parameters, and the top 10 docked poses were reserved for the binding mode analysis.

### Statistical analysis

All experiments were performed at least 3 times, and results are reported as mean ± 95% confidence intervals, unless otherwise stated. GraphPad Prism 5.0 (GraphPad Software, San Diego, CA) was used for statistical analysis. A *P* < 0.05 was considered statistically significant.

## Abbreviations

PDGFRα: Platelet-derived growth factor receptor α; (FIP1L1)-PDGFRα: FIP1-like 1-PDGFRα; CEL: chronic eosinophilic leukemia; EMSA: electrophoretic mobility shift assay; TCF/LEF: T cell factor/lymphoid enhancer factor; CHX: cycloheximide; APC: adenomatous polyposis coli; GSK3β: kinase glycogen synthase kinase 3β.

## Competing interest

The authors declare that they have no competing interests.

## Authors’ contributions

YJ designed, performed experiments and analyzed data; KD synthesized ponatinib; HL and MX performed molecular docking analysis; XS and CW performed experiments of apoptosis. JP designed, performed research, analyzed data, directed the whole study and wrote the manuscript. All authors read and approved the final manuscript.

## Supplementary Material

Additional file 1 **Computer-simulated binding of ponatinib to the native or mutated PDGFRα kinase in DFG(Asp-Phe-Gly)-out state.** The explanation was described in the fulltxt. Click here for file
