# Correction to: Ponatinib efficiently kills imatinib-resistant chronic eosinophilic leukemia cells harboring gatekeeper mutant T674I FIP1L1-PDGFRα: roles of Mcl-1 and β-catenin

**DOI:** 10.1186/s12943-021-01407-6

**Published:** 2021-10-22

**Authors:** Yanli Jin, Ke Ding, Honglin Li, Mengzhu Xue, Xiaoke Shi, Chengyan Wang, Jingxuan Pan

**Affiliations:** 1 Department of Pathophysiology, Zhongshan School of Medicine, Sun Yat-sen University, Guangzhou, China; 2 Key Laboratory of Tropical Disease Control (Sun Yat-sen University), Ministry of Education, Guangzhou, China; 3 Key Laboratory of Regenerative Biology and Institute of Chemical Biology, Guangzhou Institute of Biomedicine and Health, Chinese Academy of Sciences, Guangzhou Science Park, Guangzhou, China; 4 Shanghai Key Laboratory of Chemical Biology, School of Pharmacy, East China University of Science and Technology, Shanghai, China; 5 State Key Laboratory of Ophthalmology, Zhongshan Ophthalmic Center Sun Yat-sen University, 54 Xianlie Nan Road, Guangzhou, 510060 People’s Republic of China; 6 Collaborative Innovation Center for Cancer Medicine, State Key Laboratory of Oncology in South China, Sun Yat-Sen University Cancer Center, Guangzhou, 510060 China

**Correction to: Mol Cancer 13, 17 (2014)**

**https://doi.org/10.1186/1476-4598-13-17**

Following publication of the original article [1], minor errors were identified in the images presented in Figs. 1 and 3; specifically:

- Fig. 1d: immunoblot band for p-Erk1/2 in BaF3-T674I FIP1L1-PDGFRα cells has been replaced with the correct image
- Fig. 3e: immunoblot bands for Bim in both BaF3-WT FIP1L1-PDGFRα and BaF3-T674I FIP1L1-PDGFRα cells have been replaced with the correct images

The corrected figures are given below. The correction does not have any effect on the results or conclusions of the paper. The original article has been corrected.

**Fig. 1:**
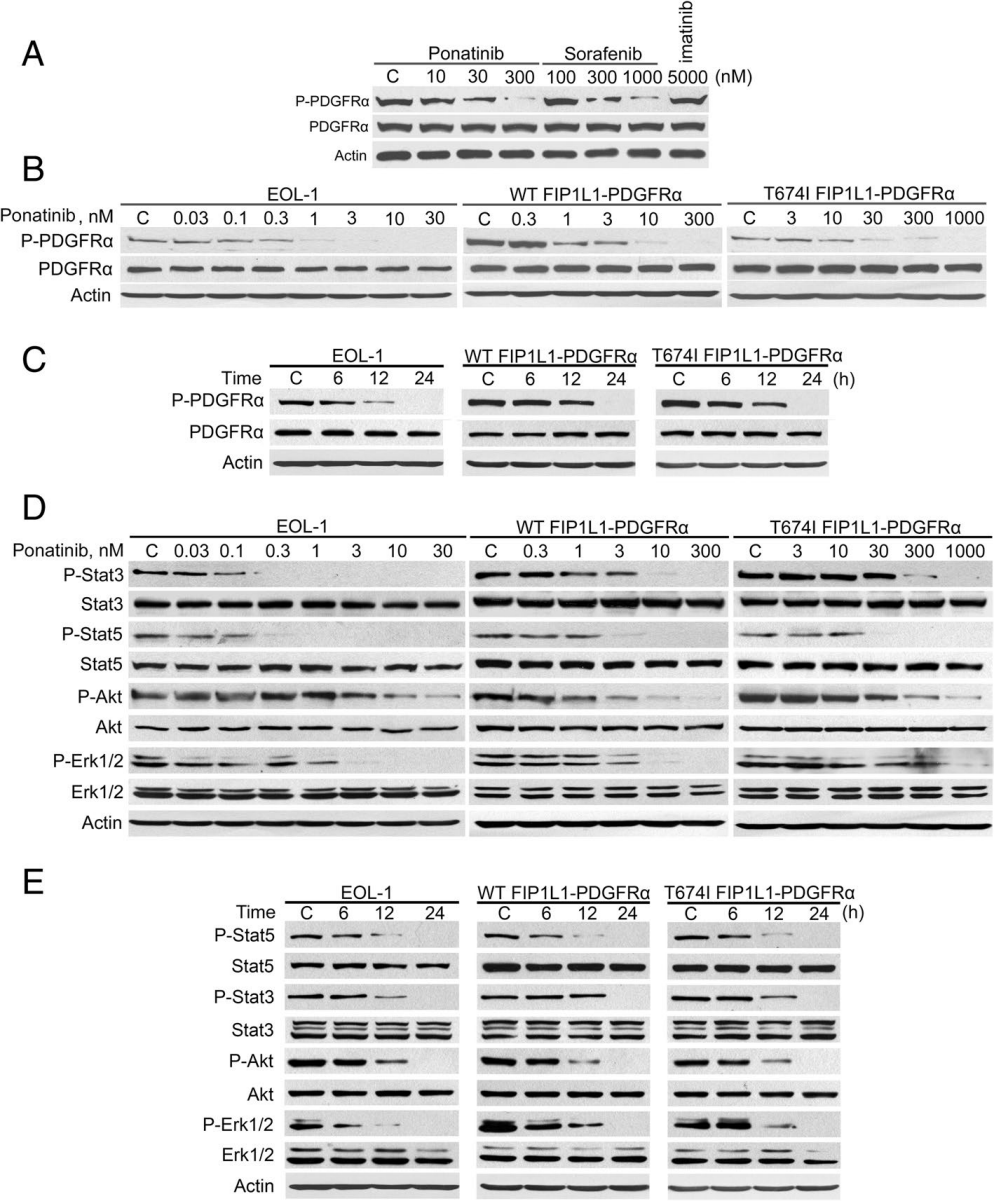
Ponatinib inhibits phosphorylation of PDGFRα and its downstream signaling molecules. **A** BaF3-T674I FIP1L1-PDGFRα cells exhibited differential sensitivity to ponatinib and sorafenib. BaF3-T674I FIP1L1-PDGFRα cells were treated with the TKIs at the indicated concentrations for 24 h, and the levels of phosphorylated and total PDGFRα were detected with the relevant antibodies. **B** Ponatinib inhibited phosphorylation of PDGFRα in a concentration-dependent manner. EOL-1 and BaF3-WT or -T674I FIP1L1-PDGFRα cells were exposed to escalating concentrations of ponatinib for 24 h. **C** Ponatinib inhibited phosphorylation of PDGFRα in a time-dependent manner. The concentrations of ponatinib were 1 nM for EOL-1, 300 nM for BaF3-WT and -T674I FIP1L1-PDGFRα cells, respectively. **D** Ponatinib concentration-dependently inhibited phosphorylation of Stat3, Stat5, Akt and Erk1/2. The cells were exposed to increasing concentrations of ponatinib for 24 h. **E** Ponatinib time-dependently inhibited phosphorylation of Stat3, Stat5, Akt and Erk1/2. 300 nM ponatinib was applied.

**Fig. 3:**
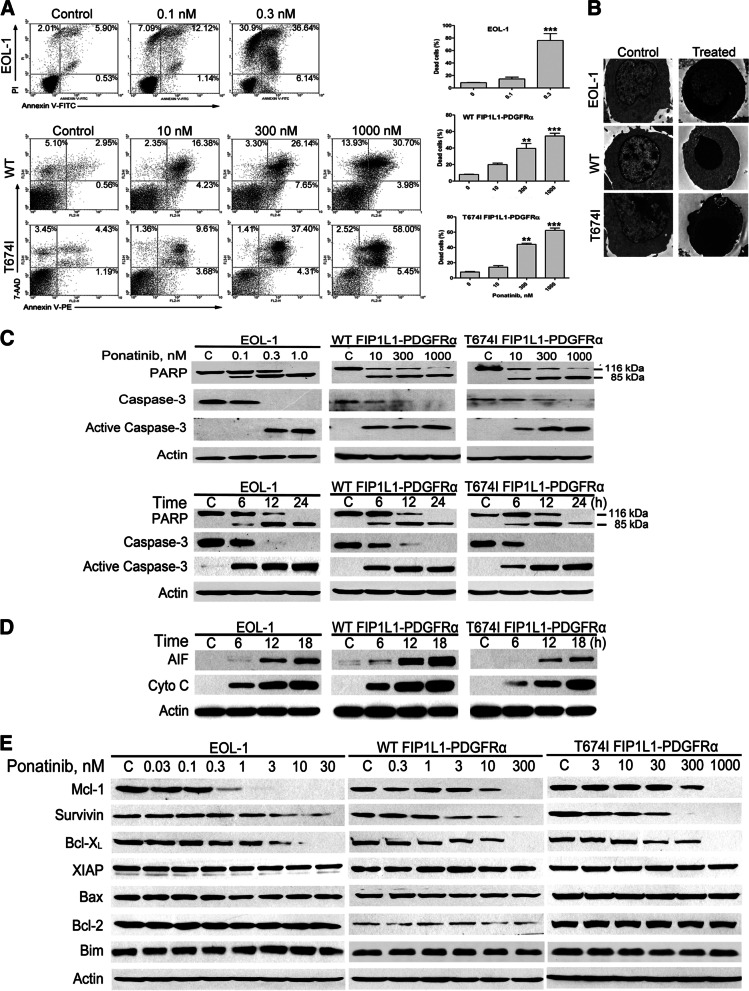
Ponatinib induces apoptosis in FIP1LI-PDGFRα-expressing cells. **A** EOL-1 and BaF3-WT or -T674I FIP1L1-PDGFRα cells were exposed to increasing concentrations of ponatinib for 24 h, apoptotic cells were assayed with flow cytometry by PI/Annexin V-FITC (EOL-1) or 7-AAD/Annexin V-PE (BaF3-WT or -T674I FIP1L1-PDGFRα cells) staining. Left, representative histograms; Right, statistical data of 3 independent experiments, the vertical axis stands for the sum of all dead cells. Error bars represent 95% confidence intervals. **, *P* < 0.01; ***, *P* < 0.0001, one-way ANOVA, *post hoc* comparisons, Tukey’s test. **B** The indicated cells were treated with or without ponatinib (1 nM for EOL-1, 300 nM for BaF3-WT and -T674I FIP1L1-PDGFRα cells, respectively) for 24 h, washed with PBS and fixed with 2% glutaraldehyde plus 2% paraformaldehyde in 0.1 M cacodylate buffer (pH 7.3). Representative photographs (9700×) were acquired under transmission electron microscopy. **C** The concentration- (for 24 h) and time-dependent (1 nM for EOL-1, 300 nM for BaF3-WT and -T674I FIP1L1-PDGFRα cells) cleavage of PARP and caspase-3 triggered by ponatinib was analyzed by immunoblotting. **D** Ponatinib elicited release of AIF and cytochrome *c* into the cytosol. Cells were treated with 1 nM ponatinib for the indicated durations and the cytosolic fraction was extracted with digitonin buffer. Levels of AIF and Cytochrome *c* (Cyto c) were detected by immunoblotting. **E** Immunoblotting of apoptosis-related proteins in CEL cells after treatment for 24 h
