# Supplementary material for: Ponatinib efficiently kills imatinib-resistant chronic eosinophilic leukemia cells harboring gatekeeper mutant T674I FIP1L1-PDGFRα: roles of Mcl-1 and β-catenin
Source: Mol Cancer. 2014 Jan 28;13:17. doi: 10.1186/1476-4598-13-17 (PMC3928078; doi:10.1186/1476-4598-13-17)
Supplement: Additional file 1 — Computer-simulated binding of ponatinib to the native or mutated PDGFRα kinase in DFG(Asp-Phe-Gly)-out state. The explanation was described in the fulltxt. [file 1476-4598-13-17-S1.doc]

**Supplementary Figure S1**


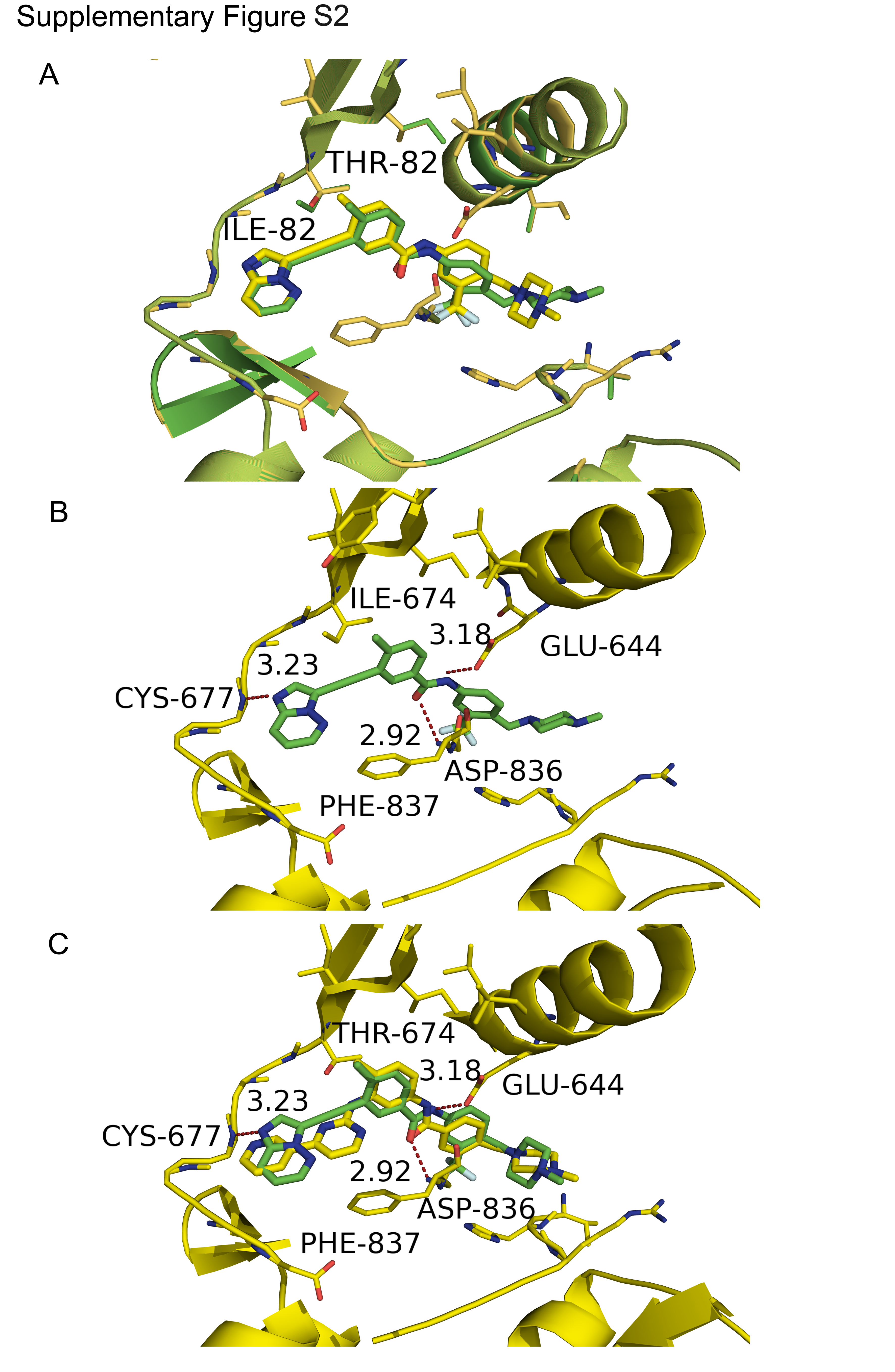


**Supplementary Fig. S1. Computer-simulated binding of ponatinib to the native or mutated PDGFRα kinase in DFG(Asp-Phe-Gly)-out state.** The explanation was described in the fulltxt.
